# Treatment of Substance Use Disorders With a Mobile Phone App Within Rural Collaborative Care Management (Senyo Health): Protocol for a Mixed Methods Randomized Controlled Trial

**DOI:** 10.2196/65693

**Published:** 2025-03-26

**Authors:** Tyler S Oesterle, Nicholas L Bormann, Margaret M Paul, Scott A Breitinger, Benjamin Lai, Jamie L Smith, Cindy J Stoppel, Stephan Arndt, Mark D Williams

**Affiliations:** 1 Department of Psychiatry Mayo Clinic Rochester, MN United States; 2 Robert D. and Patricia E. Kern Center for the Science of Health Care Delivery Mayo Clinic Rochester, MN United States; 3 Department of Family Medicine Mayo Clinic Rochester, MN United States; 4 Department of Psychiatry University of Iowa Iowa City, IA United States; 5 Department of Biostatistics University of Iowa Iowa City, IA United States

**Keywords:** substance-related disorders, mobile apps, primary care, clinical trial, substance use disorder, SUD, addiction treatment, telemedicine, telepsychiatry, care management, community-based care, behavioral health program

## Abstract

**Background:**

COVID-19 worsened an already existing problem in substance use disorder (SUD) treatment. However, it helped transform the use of telehealth, which particularly benefits rural America. The lack of specialty addiction treatment in rural areas places the onus on primary care providers. Screening, brief intervention, and referral to treatment (SBIRT) is an evidenced-based strategy commonly used in primary care settings to target SUD outcomes and related behaviors. The integration of telehealth tools within the SBIRT pathway may better sustain the program in primary care. Building on Mayo Clinic’s experience with collaborative care management (CoCM) for mental health treatment, we built a digitally native, integrated, behavioral health CoCM platform using a novel mobile app and web-based provider platform called Senyo Health.

**Objective:**

This protocol describes a novel use of the SBIRT pathway using Senyo Health to complement existing CoCM integration within primary care to deliver SUD treatment to rural patients lacking other access. We hypothesize that this approach will improve SUD-related outcomes within rural primary care clinics.

**Methods:**

Senyo Health is a digital tool to facilitate the use of SBIRT in primary care. It contains a web-based platform for clinician and staff use and a patient-facing mobile phone app. The app includes 16 learning modules along with data collection tools and a chat function for communicating directly with a licensed drug counselor. Beta-testing is currently underway to examine opportunities to improve Senyo Health prior to the start of the trial. We describe the development of Senyo Health and its therapeutic content and data collection instruments. We also describe our evaluation strategy including our measurement plan to assess implementation through a process guided by Consolidated Framework for Implementation Research methods and effectiveness through a waitlist control trial. A randomized controlled trial will occur where 30 participants are randomly assigned to immediately start the Senyo intervention compared to a waitlist control group of 30 participants who will start the active intervention after a 12-week delay.

**Results:**

The Senyo Health app was launched in May 2023, and the most recent update was in August 2024. Our funding period began in September 2023 and will conclude in July 2027. This protocol defines a novel implementation strategy for leveraging a digitally native, clinical platform that enables the delivery of CoCM to target an SUD-specific patient population. Our trial will begin in June 2025.

**Conclusions:**

We present a theory of change and study design to assess the impact of a novel and patient-centered mobile app to support the SBIRT approach to SUD in primary care settings.

**Trial Registration:**

ClinicalTrials.gov NCT06743282; http://clinicaltrials.gov/ct2/show/NCT06743282

**International Registered Report Identifier (IRRID):**

PRR1-10.2196/65693

## Introduction

Limited access to substance use disorder (SUD) treatment was a problem long before COVID-19. Approximately 22 million American people needed SUD treatment in 2019, but only 13% received any intervention [1]. The COVID-19 pandemic coincided with an increase in SUD prevalence, as treatment programs were restricted or closed [2]. The National Institutes of Health described the compounding effects of the concurrent opioid epidemic and COVID-19 pandemic as a national emergency [3]. In response, the White House Office of National Drug Control Policy recommended increased funding for telehealth services and mobile phone apps.

Telehealth use has increased dramatically since the COVID-19 pandemic onset [4]. While numerous patients may benefit from its flexibility, rural patients and stigmatized populations, such as individuals with addiction, may be particularly aided [5]. Before COVID-19, rural patients already had fewer health care providers and mental health service offerings [6]. The pandemic was associated with an increased incidence of SUD in rural areas, where isolation, stress, and boredom exacerbate risks to psychological health [7]. The shortage of SUD providers impairs individuals from receiving specialty treatment and places increased pressure on primary care providers [8]. The incorporation of telehealth helps bridge this gap. We recently published a review showing that the synchronous delivery of care through video visits for SUD treatment has robust safety and efficacy evidence [2].

Screening, brief intervention, and referral to treatment (SBIRT) is the most common strategy to address problematic substance use in primary care. However, there have been significant barriers to translating it into treatment. Data combined from the 2015 to 2019 National Survey on Drug Use and Health found that only 69.9% of adults with alcohol use disorder were screened for alcohol use, with this leading to approximately 5% to 6% of adults who might benefit from treatment being referred to and eventually receiving treatment [9]. Known barriers to SBIRT use are limited clinician time, competing clinical priorities, providers’ belief that the intervention will be ineffective, lack of training and experience with delivering the intervention, and the need for integration with the addiction care delivery model [10]. These factors can be addressed with adequate preimplementation support and training [11].

Collaborative care management (CoCM) is a system-based approach that integrates behavioral health care managers into primary care clinics with the support of psychiatrists to assist primary care providers in managing mental health disorders. This improves treatment access and outcomes while decreasing the need for independent psychiatric services [12]. Current evidence supports case management services delivered by various backgrounds (nursing, welfare or social workers, and mental health therapists) to strengthen service connections [13,14]. CoCM has demonstrated effectiveness in improving SUD treatment access, increasing resource efficiency, and reducing substance use [15-17]. However, addressing SUDs within CoCM poses several challenges, including organizational process and infrastructure capacity development for CoCM implementation, training behavioral health care managers, accessing specialty addiction consultation, a national shortage of SUD providers, and overcoming staff stigma [18]. CoCM also relies on symptom monitoring tools [12], and when used within addiction treatment, these must comply with federal and state confidentiality and consent regulations.

Digital CoCM adapts the traditional CoCM framework to a digital format, leveraging telemedicine platforms to overcome geographical and logistical barriers to care. Unlike traditional CoCM, which relies on in-person care teams embedded in clinics, digital CoCM uses digital tools to facilitate integration and communication between primary care providers, care managers, and consulting psychiatrists. Digital platforms allow for the integration of structured workflows, enabling asynchronous communication between providers and giving real-time updates on patient progress. This approach enhances flexibility while maintaining the core components of CoCM: care coordination, systematic case review, measurement-based care, and patient-centered outcomes [19].

Compared to stand-alone telehealth services or other SUD-focused apps, digital CoCM emphasizes integration across the care team rather than focusing solely on the patient-provider dyad. While telehealth visits allow for synchronous patient-provider interactions, they may lack systematic tracking of patient progress, team-based coordination, or integration of psychiatric consultation. SUD-focused apps typically provide self-management tools, such as cognitive behavioral therapy (CBT) modules or contingency management (CM), but they often operate outside the clinical care team and do not facilitate collaborative decision-making between primary care providers and care managers [20]. Digital CoCM bridges this gap by embedding app-based tools within a team-based care model, ensuring that technology supports both patient engagement and provider collaboration. Digitally delivered interventions can positively augment the patient-provider experience by incorporating new ways to interact [21,22], enabling the easy monitoring of clinically relevant measures of substance use and mental health symptoms, and supporting evidence-based practices. However, to our knowledge, no digital telemedicine platform has been tested to facilitate integrated SUD CoCM within primary care. This study aims to evaluate the implementation and clinical effectiveness of Senyo Health, a digitally native, addiction treatment and CoCM platform designed to address barriers to SUD treatment in rural primary care settings. Specifically, we aim to identify facilitators and barriers to implementation; improve the integration of SUD treatment into primary care workflows; and assess the platform’s impact on substance use outcomes, treatment retention, and recovery trajectory. We hypothesize that this intervention will reduce the frequency and intensity of substance use, promote addiction recovery, and be well received by primary care staff. This protocol details the iterative design, development, pilot-testing, and randomized controlled trial study stages.

## Methods

### Study Design

**Figure 1:**
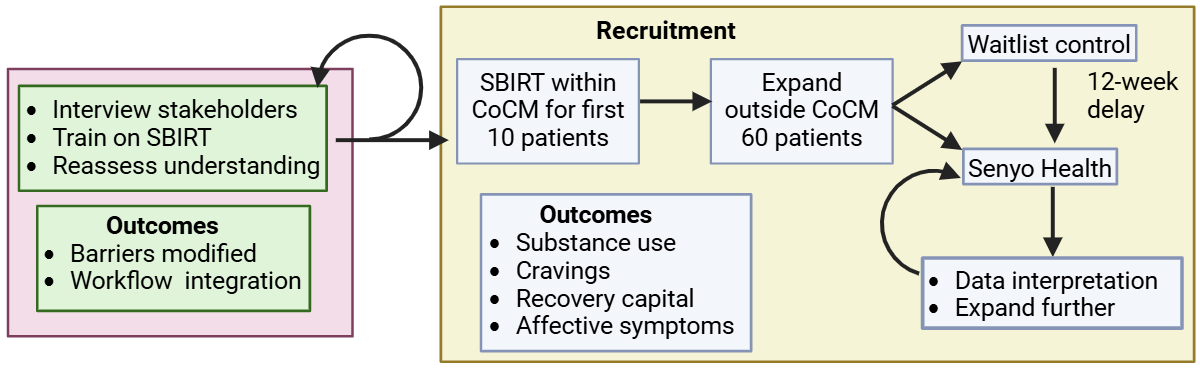
Study flow diagram. The study is an exploratory, sequential, mixed methods design. The initial study phase is qualitative (left, pink square) with a focus on interviewing clinical stakeholders (physicians, nursing, and medical support staff), training on SBIRT, and reassessing their understanding and experience to iteratively improve the process. The second study phase is quantitative (right, yellow rectangle) with outcomes noted. The first 10 patients will be recruited from ongoing CoCM for mental health services after screening positive on validated alcohol and drug use measures. We then will recruit an additional 60 patients, who will participate in a randomized controlled trial of a 12-week intervention with Senyo Health (active phase) or to a 12-week waitlist control group who will subsequently receive the active intervention. These results and feedback will be used to iteratively improve the intervention after study completion. CoCM: collaborative care management; SBIRT: screening, brief intervention, and referral to treatment.

This is a mixed methods study. The first stage is focused on implementing SBIRT into primary care and identifying barriers. During this stage, we will use Consolidated Framework for Implementation Research (CFIR) [23]. The CFIR’s core domains include intervention characteristics, which focus on the attributes of the intervention itself, such as its complexity and adaptability. The outer and inner settings examine the external and internal contexts, including factors like patient needs, organizational culture, and readiness for implementation. Finally, the characteristics of individuals and the process domains address the roles of individuals involved and the steps taken to implement the intervention, such as planning and engaging stakeholders. The second stage is a waitlist randomized controlled trial studying the effectiveness of the Senyo Health platform in decreasing substance use over a 12-week period (Figure 1). The intervention will have 4 core components that will be iteratively improved based on stakeholder feedback. First, each patient will receive digitally native CoCM for SUD with weekly web-based check-ins with licensed alcohol and drug counselors (LADCs), who will be the main point of contact for patients enrolled in the study. Consistent with CoCM, standardized symptom monitoring and monthly reviews of all clinical cases will occur with the LADC and the overseeing psychiatrist. Second, the patient will be offered, initiated, or continued on medications to manage psychiatric and addiction diagnoses and symptoms. Third, the patients will use a digital clinical platform to complete a set of CBT and behavioral activation–based psychotherapeutic modules. Fourth, patients will use a digital CM platform through an app on their phones.

### Ethical Considerations

This study was reviewed and approved by the Mayo Clinic Institutional Review Board (IRB) for human research (#24-007758) in accordance with institutional policies and federal regulations governing human participant research. The IRB determined that this study requires full board review, and no exemption or waiver was granted. Informed consent will be obtained directly from all participants prior to study enrollment, and participants will have the ability to opt out at any time without consequence. This study does not involve secondary data analysis requiring additional consent considerations. No identifiable patient information will be reported, ensuring participant confidentiality and data security. Data collected by the app system are securely transferred using industry-standard encryption to the “Backend Services” (ie, Mayo Clinic servers behind Health Insurance Portability and Accountability Act [HIPAA]–compliant firewall). This cloud-based infrastructure serves and communicates with the patient-facing app. The Backend Services contain all data and analytics specific to the app clients (participants, researchers, and providers). No protected health information is contained in the app itself. All data used by the app follow internationally recognized security standards, including the National Institute of Standards and Technology SP800-53 and HIPAA. All patient information is automatically encrypted when entered in the system, allowing for secure data transfer from the participant device to the provider interface and storage. The treatment phase of this project uses the CM method. Participants earn points for completing treatment tools within the Senyo app. They turn in those points for rewards of specified dollar amounts. These dollar amounts are loaded on a reloadable cash card (ClinCard). The app will notify study staff of the dollar amount, which will be loaded onto the card and documented in the research participant remuneration application system. The amount of points a participant can earn is approximately 1400 (converts to US $350), but this amount will vary depending on participation and points received. In the follow-up and waitlist phase, participants will receive US $25 for each visit where they complete interviews and questionnaires. Those assigned to the waitlist phase will have an additional 12 weeks of participation than those assigned to the intervention phase. Remuneration will not be provided during time on the waitlist.

### Senyo Health Intervention

#### Overview

Senyo Health has several core features that enable it to deliver digital CoCM for SUD treatment. Senyo offers several well-studied, app-based components to users, including asynchronous CBT modules, CM, behavioral activation, and an interface to interact with the program LADC. We chose these features as our initial intervention based on our research that showed CBT and CM had significant effect sizes for improving substance use-related outcomes, with CM having a large effect [20]. A group of licensed drug counselors meeting an hour a week over the course of several months with a senior patient education specialist employed by Mayo Clinic took individual paper copies of patient education content that presented CBT-based concepts and combined them with CBT content found in National Institutes of Health CBT Content built for “Project MATCH” [24]. This process resulted in the production of 16 modules.

Traditional care coordinator and patient coordination visits involve a comprehensive assessment of the patient’s physical, mental, and social health needs. It includes collaborative care planning with input from various health care providers, patient education, and regular follow-up. These visits are typically done in person or over the phone. To add digital care coordination, we incorporated the ability to communicate directly with a LADC care coordinator through the app. Furthermore, the Senyo platform enhances care coordination by providing the care coordinator with a desktop portal directly linked to the patient’s mobile phone app. The care coordinators have immediate access to the patient’s progress through an interface that showed the patient’s use of the mobile phone app features, survey results, and activities. The platform also allows the care coordinator to do video-based conversations that more closely mimic in-person care coordination visits.

**Figure 2:**
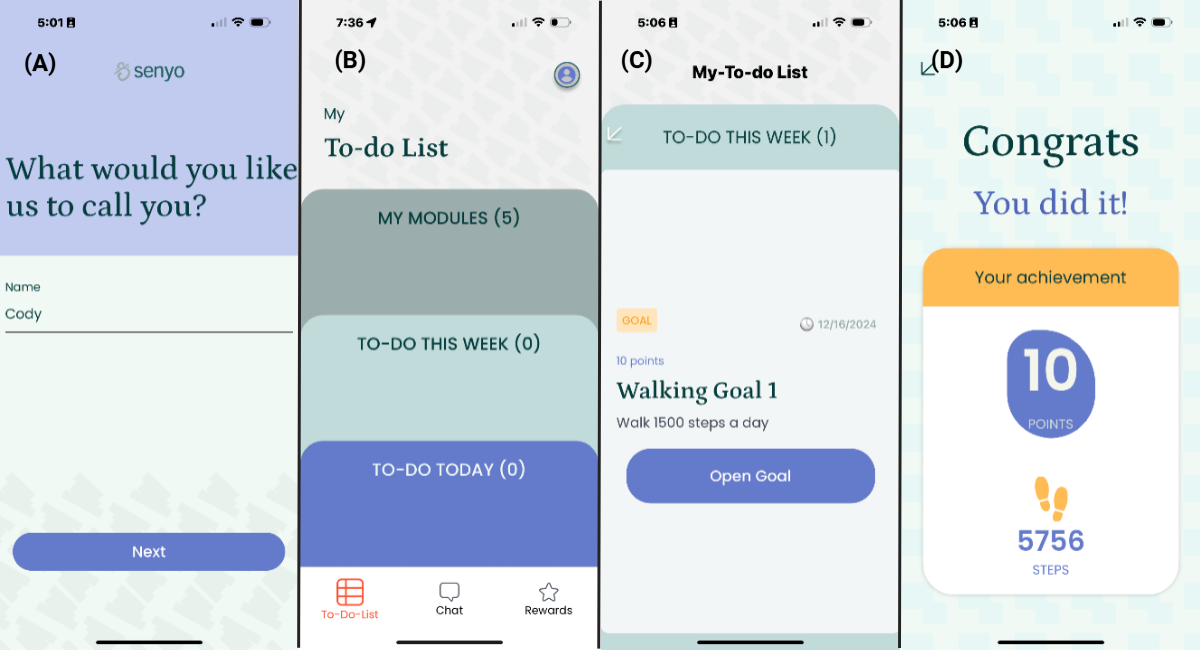
Screenshots from Senyo Health app that the individual using the app sees. Panel (A) shows the initial personalization. Panel (B) shows the current “To-do List” and also displays the chat icon at the bottom of the panel. Panel (C) shows a behavioral activation task, and panel (D) showcases the points awarded for completing this task.

**Figure 3:**
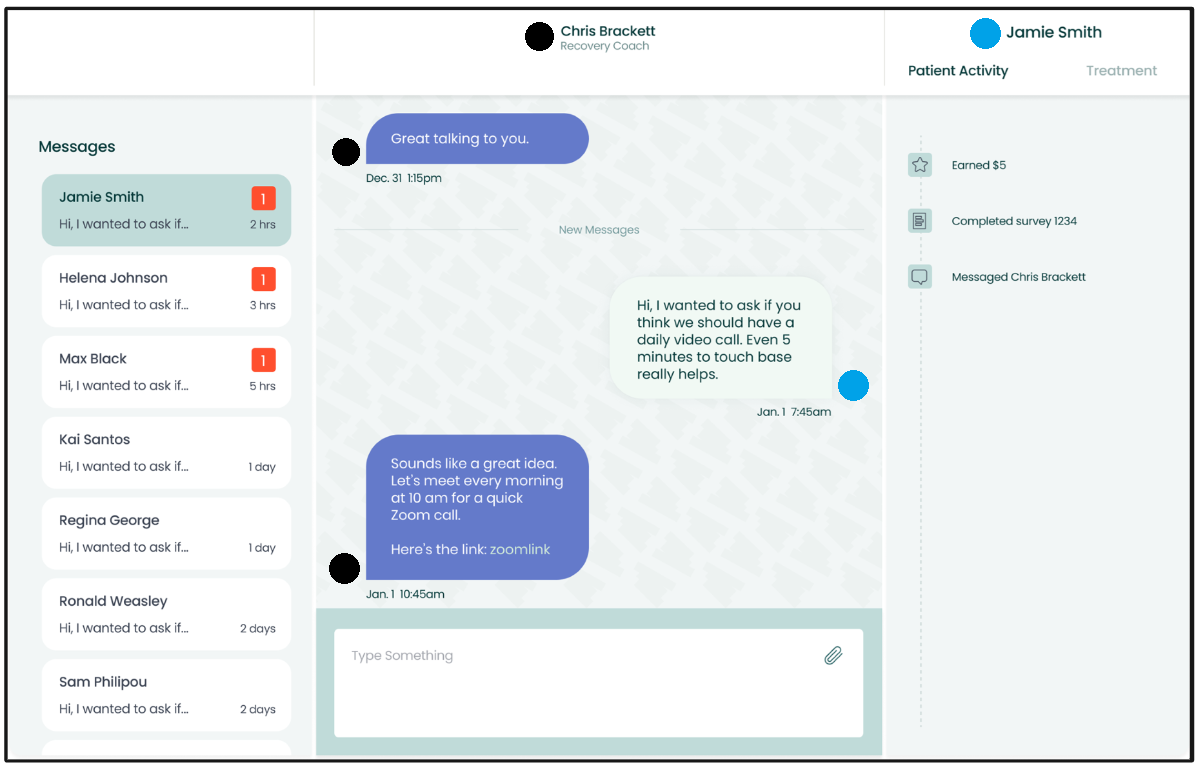
Senyo Health chat feature being displayed from the perspective of the recovery coach. The left is conversations with multiple patients. Once selected, the full conversation appears in the center of the screen, with the recovery coach able to text back and forth. Surveys, modules, and activation tasks can also be assigned to the participant through the chat.

The app was designed to be simple and user-friendly. When first using the app, initial prompts are designed for the user to create personalization and review security or privacy settings. Figure 2 displays the patient’s to-do list, a behavioral activation goal (walking), and the points received for completing that goal. Figure 3 displays the chat feature, which is a communication portal with the LADC. Modules and surveys can be assigned in response to current symptoms in real time.

#### CBT Modules

Apps allow for the asynchronous delivery of therapeutic content that participants can use at any time. Current research supports delivering asynchronous content through “modules” or “activities.” Psychotherapeutic modules within apps contain brief text-based content, videos, and related questions that patients can experience independently whenever convenient. CBT is the most researched content type for asynchronous modules [15,25,26]. “Activities” within apps are usually actions that the patient performs, such as filling out a survey, doing a mindfulness exercise, or being physically active [27]. Senyo Health currently contains 16 modules with content based on Mayo Clinic patient education pamphlets. Topics include “addictive thought patterns,” “living with emotions in recovery,” “self-esteem and relationships,” “my recovery building blocks,” “self-care in recovery,” “cravings,” and “relapse prevention.”

#### CM Component

CM is a behavioral therapeutic intervention where monetary or prize-based rewards are “contingent” on objective evidence of drug abstinence and abstinence-promoting behaviors, such as attending mutual support meetings and counseling sessions [28]. Therefore, CM does not deliver education or concepts for individuals to learn; instead, it is a strategy to encourage positive behaviors. CM has decades of research representing hundreds of controlled trials demonstrating its safety and efficacy in assisting in-person SUD treatment [28,29]. It is arguably one of the most effective therapeutic strategies available to date but can be challenging to implement within conventional in-person treatment programs [30]. “ReSET” and “ReSET-O” are Food and Drug Administration–approved products that are available for prospective patients through a prescription that use CM to encourage the completion of in-app modules [25,26]. Digitally delivered CM through an app appears to improve engagement at a similar level to CM delivered in person [31].

The incorporation of CM-based token economy architecture into a CoCM platform makes Senyo Health unique [32]. The app awards points for completing surveys, modules, and activities. The points per action are customizable through the research interface, allowing for the adjustment of relative incentives associated with each task. Participants can use points to purchase rewards through a reloadable card (ClinCard). The purchased rewards will be tracked and documented in the research participant remuneration application. For this study, the point value is US $0.25 per point. One point is earned for each page of a module, minute of a video, and question in a survey.

#### Activity or Goals

Senyo Health was built to work with Apple and Google health kit wearable platforms to gather data from wearable device sensors. If a device is detected and the patient authorizes this, the wearable device sensor will track activity, heart rate, and oxygen saturation. These data will be used to encourage physical activity by connecting sensor data to CM rewards. These data will only be collected from participants who already have and wear a personal device; the study will not provide such device to participants.

#### LADC to Patient User Interface

The LADC will use the provider portal to communicate through the app with their patients. Social rewards through apps, such as support from a clinician or peer via messaging or telephone, produce significantly greater user engagement than fully automated apps [32]. Typically, this support aims to maintain patient adherence with the app, monitor user progress through periodic symptom assessments, assist the patient in understanding therapeutic concepts or skills training, and triage patients who do not respond to app-based interventions [33].

### Study Sample and Recruitment

#### Overview

The implementation phase will start with a pilot within a rural primary care site in the upper Midwest region of the United States that is familiar with the CoCM model for mental health treatment. After following the implementation strategy below at the initial location, we will expand to 2 additional primary care sites—1 for a rural service population and 1 for a nonrural service population. This staged rollout will allow for iterative improvements for the subsequent sites based on knowledge gained from the first site.

#### Provider Engagement and Implementation

##### Preimplementation

Clinic engagement efforts will start by recruiting a primary care champion at the primary care site. Other primary care clinic key stakeholders (physicians, nursing, CoCM staff, and medical support and administrative staff) will be included as appropriate. Focus groups and interviews with providers and staff engaged in the SBIRT workflow will assess attitudes and perceived barriers toward SBIRT, the use of medications for addiction treatment (buprenorphine, acamprosate, etc), and the use of digital delivery of SUD treatment within CoCM. Focus groups will be interviewed using a semistructured interview based on the CFIR interview guide. Please see Multimedia Appendix 1 for details.

##### Implementation: Training, Supervision, and Refinement

Research team members with experience treating SUD in primary care clinics will provide education to providers at the participating site. We will be scheduled as part of a regularly recurring staff education curriculum to provide didactic base education on the project (specifically the screening metrics, conducting a brief intervention, and how to refer to this study). The LADC will support voluntary follow-up training on brief interventions, motivational interviewing, and the referral process. Primary care champions at each participating site will encourage other site providers to attend training and initiate medications for addiction treatment for patients with confirmed moderate and severe SUD diagnoses. These champions will also liaise with the LADC.

Throughout the implementation approach described, we will gather feedback from the primary care team on SBIRT, the Senyo Health platform, the CoCM team, and LADC to evaluate the processes. Primary care providers, nurses, key administrators, and clinic staff from each of the sites will be identified for interviews. Feedback on these components will be used to iterate and refine our strategy.

#### SBIRT Protocol

##### Screening

Universal patient screening with the Drug Abuse Screening Test (DAST) [34] and the Alcohol Use Disorders Identification Test-Consumption (AUDIT-C) [35] is an eventual goal. These instruments help determine the need for treatment referral using the SBIRT care pathway. The AUDIT-C is already frequently implemented across primary care; however, the DAST is not. Screening frequency will be explored in the feasibility stages based on feedback from clinic staff and leadership. While the US Preventive Services Task Force recommends all adults are screened for substance use, this may not be feasible in all clinic workflows. Stakeholder feedback will monitor excessive screening burden, and process iteration will attempt to minimize unnecessary rescreening to optimize overall results.

##### Brief Intervention

The brief intervention stage is where the SBIRT pathway often stalls out. Implementation of a brief intervention will be collaborative and iterative with the initial pilot site. FRAMES [36], which consists of feedback, responsibility, advice, menu for change, empathy, and enhancing self-efficacy, will be the primary component of this intervention. If granted permission by the patient in the screening stage, a LADC will provide a brief intervention to the patient (ie, personalized feedback, education, and motivational interviewing). The primary care provider is at liberty to provide some initial counseling to the patient, but the full FRAMES-based structure of the brief intervention is deferred to the LADC. In this modification of the traditional SBIRT framework, the goal is to minimize the additional clinical burden on the primary care providers and emphasize the value of the CoCM program in augmenting the capabilities and capacities of the primary care team. The primary goal of the brief intervention as conducted by the LADC is to facilitate patient enrollment in CoCM or referral to a higher level of care if needed.

##### Referral

Patients will be informed about local treatment options for treatment as usual and about the option to participate in this study by enrolling in the Senyo CoCM program. They will be allowed to choose which option they feel is best for them. Once the process runs smoothly at the pilot site, we will expand to the next selected site.

##### Treatment

All patients who consent to study participation will receive care through the Senyo Health platform. However, randomization will determine if this is provided immediately or if access is delayed by 12 weeks (ie, waitlist design). These patients will have outcomes closely monitored as described in the Study Procedures section. To ensure all eligible patients receive treatment regardless of trial participation, study coordinators will attempt to follow up with those who chose treatment as usual to monitor if they do ultimately receive treatment.

##### Study Criteria

The intervention will be piloted to 10 patients who screen positive on the DAST or AUDIT-C and are already participating in CoCM for depression within primary care (no randomization). In addition to screening results, the clinical team will use their clinical judgment to determine eligibility, prioritizing patients for whom a SUD appears to be the primary psychiatric diagnosis rather than a mood or anxiety disorder. Once these patients have been recruited effectively and we have solidified our implementation strategy, we will broaden screening to primary care patients not currently in CoCM at the participating site. An additional 60 patients will be randomly assigned to immediate access to Senyo Health versus the waitlist. The 30 participants randomly assigned to the waitlist arm will be later added to the active arm (Senyo Health platform), for a total of 70 patients receiving active intervention by study conclusion. A computerized random assignment list will be generated by the study statistician for the study coordinator to assign enrollees sequentially, stratified by sex and restricted to every 10 cases, ensuring equal numbers per group. Due to the nature of the intervention and trial design, no one will be blinded. The study will recruit patients from Mayo Clinic primary care clinics at 3 sites after they have agreed to participate, with 2 serving primarily rural patients and 1 serving primarily nonrural patients.

Inclusion criteria are as follows: age 18 years and older; ability to read, write, and understand English; minimum DAST (1+) or AUDIT-C (3+) scores; and access and willingness to use a mobile device for asynchronous (text) and synchronous (video visits) engagement with care. Exclusion criteria are as follows: already participating in or about to initiate treatment in another structured addiction treatment program; diagnosed personality pathology as the primary presenting concern based on clinical judgment, severe cognitive impairment (eg, intellectual disability or dementia), or psychosis; inability to actively participate in and learn from psychotherapeutic interaction based on clinical evaluation and clinical judgment; needing a higher level of mental health care as demonstrated by American Society of Addiction Medicine assessment; and decline to answer suicidality questions.

### Study Procedures

#### Overview

Once primary care patients have selected the study treatment path, the steps outlined below will occur. These include obtaining and signing informed consent, collecting medical and psychiatric history for comorbidities through patient interview, and answering validated questionnaires on mood, anxiety, and their overall strengths and resources to achieve substance use cessation (ie, recovery capital). Recovery capital is a biopsychosocial model of recovery from addiction, which encapsulates the interpersonal strengths and resources an individual can leverage in pursuit of substance use cessation [37]. Next, participants will be randomly assigned to the waitlist or active intervention. Once randomly assigned, participants will complete visits through a clinical treatment phase and a follow-up phase. The waitlist group will serve as a “waitlist” control and be asked to “cross over” and receive the active intervention after 12 weeks on the waitlist.

Study activities are listed in Textbox 1 and detailed in Table 1.

**Study activities.**

- Baseline visit
  Obtain informed consent from the participant.
  American Society of Addiction Medicine diagnostic interview [38] conducted by a licensed alcohol and drug counselor (LADC).
    - Participants must meet the criteria for outpatient treatment or indicate that outpatient is their preference.
  Questionnaires: Brief Substance Craving Scale [39]; Brief Assessment of Recovery Capital [40]; self-reported substance use based on the Timeline Followback [41]; and psychiatric comorbidities measured via Generalized Anxiety Disorder-7 [42], Patient Health Questionnaire-9 [43], and Epworth Sleepiness Scale [44].
  Treatment attendance monitoring questionnaire.
  Urine drug test—check for certain drugs, including alcohol and illicit substances (ie, cocaine and methamphetamine).
  Randomization to active intervention versus waitlist arms.
  Set up active participants in the Senyo Health app.
- 12 weekly visits with LADC and pertinent study staff during the clinical treatment phase.
  LADC will provide clinical counseling. The total number of counseling visits will be up to the discretion of the LADC, with a target goal of at least 1 hour weekly.
  Questionnaires: Brief Substance Craving Scale [39], Timeline Followback [41], Generalized Anxiety Disorder-7 [42], Patient Health Questionnaire-9 [43], and Epworth Sleepiness Scale [44].
- Additional items monthly on weeks 4, 8, and 12.
  Urine drug screen.
  Brief Assessment of Recovery Capital [40].
- Final visit of active treatment (week 12).
  Acceptability of Intervention Measure.
  Intervention Appropriateness Measure.
  Feasibility of Intervention Measure.
  System Usability Scale [45].
- The follow-up phase begins immediately after completion of 12 weeks of active treatment. Visits will occur at weeks 4, 8, and 12, and at 1 year.
  Questionnaires: Brief Substance Craving Scale [39], Timeline Followback [41], Generalized Anxiety Disorder-7 [42], Patient Health Questionnaire-9 [43], Epworth Sleepiness Scale [44], and Brief Assessment of Recovery Capital [40].
  Urine drug screen.
- Individuals randomly assigned to the waitlist phase will follow the “follow-up phase” schedule during their 12 weeks prior to active intervention.

**Table 1:** Study activities^a^.

Assessment	Baseline	Active treatment phase (week)												Follow-up or waitlist phase (week)													1 year
		1	2	3	4	5	6	7	8	9	10	11	12	1	2	3	4	5	6	7	8	9	10	11	12		
Consent	✓																										
Check-in		✓	✓	✓	✓	✓	✓	✓	✓	✓	✓	✓	✓				✓				✓				✓	✓	
TLFB^b^	✓	✓	✓	✓	✓	✓	✓	✓	✓	✓	✓	✓	✓				✓				✓				✓	✓	
BSCS^c^	✓	✓	✓	✓	✓	✓	✓	✓	✓	✓	✓	✓	✓				✓				✓				✓	✓	
ESS^d^	✓	✓	✓	✓	✓	✓	✓	✓	✓	✓	✓	✓	✓				✓				✓				✓	✓	
PHQ-9^e^	✓	✓	✓	✓	✓	✓	✓	✓	✓	✓	✓	✓	✓				✓				✓				✓	✓	
GAD-7^f^	✓	✓	✓	✓	✓	✓	✓	✓	✓	✓	✓	✓	✓				✓				✓				✓	✓	
TAM^g^	✓				✓				✓				✓				✓				✓				✓	✓	
Urine test	✓				✓				✓				✓				✓				✓				✓	✓	
BARC^h^	✓				✓				✓				✓				✓				✓				✓	✓	
SUS^i^													✓														
AIM^j^													✓														
IAM^k^													✓														
FIM^l^													✓														

^a^Participants will be randomly assigned to immediately start the 12-week study intervention or be put on a waitlist for 12 weeks and then start the study intervention. There will be long-term follow-up at 1 year.

^b^TLFB: Timeline Followback.

^c^BSCS: Brief Substance Craving Scale.

^d^ESS: Epworth Sleepiness Scale.

^e^PHQ-9: Patient Health Questionnaire-9.

^f^GAD-7: Generalized Anxiety Disorder-7.

^g^TAM: treatment attendance monitoring.

^h^BARC: Brief Assessment of Recovery Capital.

^i^SUS: System Usability Scale.

^j^AIM: Acceptability of Intervention Measure.

^k^IAM: Intervention Appropriateness Measure.

^l^FIM: Feasibility of Intervention Measure.

#### Outcomes

This protocol has 2 phases. For the initial implementation phase, the primary outcome will be a thematic analysis of coded structured interviews of clinic stakeholders, with a focus on whether noted SBIRT barriers were effectively modified during implementation. For the clinical trial phase, the study end point is the week 12 assessment of the primary outcome measure, overall substance use as measured by Timeline Followback. We anticipate that patient involvement in the active phase will lead to a significant reduction in substance use relative to the end of the waitlist period. Secondary outcomes will explore abstinence rates, treatment retention, along with treatment effects on craving, recovery capital, psychiatric well-being (Patient Health Questionnaire-9, Generalized Anxiety Disorder-7, and Epworth Sleepiness Scale), and use of clinical services throughout the intervention and follow-up periods.

#### Data Analysis

Qualitative interviews will be recorded, transcribed, and coded on a rolling basis using a rapid, template-based approach derived from the interview protocols [46]. Quantitative clinical treatment outcomes will be analyzed by comparing participant data at baseline, weekly during the intervention, and 12 weeks after the treatment (after completion of LADC visits). Our recent meta-analysis indicated that CM delivered through an app had a large effect size for improving substance use–related outcomes relative to control conditions [20]. Using this to inform our power calculation, there is greater than 0.80 power to see a large effect size between active and waitlist groups at the primary study end point. We will use information from this pilot to inform sample size calculations for a larger controlled efficacy trial. Several exploratory end points (eg, anxiety and depression) will be evaluated to contextualize the primary end-point effect. We will use likelihood methods (eg mixed models) that allow for data that are missing at random, so we will not impute any missing data. Intention-to-treat will be used.

#### Safety Monitoring

During the study, the LADC will be the first point of contact with the patient. Their app-based communications with the patient will focus on assessing symptoms, addressing clinical concerns, and providing brief encouragement to continue predetermined goals. The LADC will meet with the study psychiatrist and review the history obtained from the patient during their initial evaluation. The LADC will write a formal weekly update note on each patient, and monthly clinical tracking scores will be reviewed and reported to the consulting psychiatrist. Psychiatric recommendations for the management of addiction concerns and related mental health issues (ie, worsening mood or suicidality) will be reported back to the primary care providers, who will ultimately remain responsible for clinical decision-making and the treatment plan that may involve starting medications as a part of their clinical care. This patient review and consultation system will be managed through the provider portal, which will also serve as the patient population registry for quantitative tracking of clinical concerns with specific and quantitative end-point treatment goals.

Once a patient is enrolled in the study, the LADC and study staff will use the American Society of Addiction Medicine criteria to determine ongoing appropriateness for the current level of care [38]. To stay in the study, participants must remain eligible for outpatient SUD care. If the participant’s American Society of Addiction Medicine criteria worsens enough that psychiatric recommendation is a higher level of care, they will be referred clinically to residential SUD treatment. The participant’s active treatment phase will be stopped and moved into the follow-up phase for the schedule of visit. Adverse events will be reported to the psychiatrist for evaluation and reviewed for reporting requirements to the IRB.

## Results

The Senyo Health app was launched in May 2023, and the most recent update was in August 2024. Our funding period began in September 2023 and will conclude in July 2027. This protocol defines a novel implementation strategy for leveraging a digitally native, clinical platform that enables the delivery of CoCM to target an SUD-specific patient population. Our trial will begin in June 2025.

We have not yet started enrollment in the primary study. In December 2024, focus group testing of the mobile app with people who have SUDs and are in treatment was completed. LADCs who reviewed the mobile and desktop apps were also interviewed. Their feedback has been used to improve the interface, design, and fix bugs, preparing for the primary study enrollment.

## Discussion

### Overview

This protocol builds from the large literature base that has described the positive effects of telehealth both before and after the COVID-19 pandemic [2,47]. Most treatment-seeking individuals report high patient satisfaction with telehealth [47]. Specific to alcohol use, digitally delivered interventions have been shown to significantly decrease heavy and binge drinking for both male and female patients and increase treatment retention at 1 year [48,49]. The use of telehealth among Medicare beneficiaries with an opioid use disorder was significantly associated with greater treatment retention and reduced odds of medically treated overdose [50]. App engagement, however, is typically low for traditional health-related apps. Individuals often use these apps for less than 1 week [31]. User time, effort, and attention can measure user engagement [51,52]. Greater engagement has been correlated with improved abstinence rates among apps incorporating CM and CBT modules [53]. Social rewards through apps, such as support from a health care provider via messaging, also produce significantly greater user engagement than fully automated apps [32]. These are core features of Senyo Health and help differentiate it from other interventions.

Additionally, the use of digital interventions to drive changes in recovery capital is largely unknown. A study of individuals engaging through a recovery-based web page noted that positive digital interactions help bind individuals within groups, supporting the overall recovery process [54]. This is reflecting community involvement and social connectedness, which are important aspects of recovery capital. Little else is known about the use of digital interventions in this space.

It is important to note that app creation is expensive. Additionally, apps are slow to create and typically costly to update. Senyo Health was created with sustainability in mind. It is owned solely by the Mayo Clinic, is fully operational, and is actively in use. New modules can be created within a desktop-based application directly linked to the app that we call the “researcher portal.” The researcher portal allows for easy and rapid creation of new content without the need for changes to the app’s code or new versions of the app to be pushed to the App Store. This allows for easy iterative improvements to existing content with no development costs. This feature dramatically reduces long-term costs and allows for real-time improvements based on user feedback.

### Principal Findings

We will use mixed methods (interviews and questionnaires or scales) to evaluate implementation outcomes. We anticipate the identification of facilitators and barriers for the successful implementation of SBIRT and Senyo Health within a rural primary care workflow. Based on these findings, our hope is that through iterative enhancements, the rollout from site 1 to sites 2 and 3 will be increasingly streamlined.

While engaged in treatment services, substance use–related outcomes typically improve overall. Our proposed program is a combination of multiple evidence-based interventions, and as such, we expect a significant improvement in the active arm versus the waitlist control group. Additionally, we anticipate seeing recovery capital growth as individuals are engaged in treatment. As this growth has been significantly associated with decreased cravings [55] and past 30-day abstinence of alcohol and methamphetamine use [56,57], we hypothesize a significant correlation between substance use and recovery capital changes. As recovery capital growth for female patients has been reported as suppressed compared to male patients while in treatment [58], we will also explore possible sex differences.

### Limitations

As with all pilot trials, there are potential barriers. This intervention relies on wireless internet or cellular data capabilities. Access to these is improving across the United States; however, residents in rural areas, which is our main target population, have disparities in internet access [6,59]. We do not anticipate that this will affect the app, as the media is cached or can be buffered; however, it may negatively impact live counseling sessions. This will need to be monitored and will be individual-specific. The study also relies on the willingness of primary care patients to participate in a novel addiction treatment paradigm. We suspect that the flexibility, convenience, and potential receipt of positive reinforcers through CM will aid in recruitment. Additionally, because patients in primary care often have an unmet need for specialty addiction treatment, we anticipate strong interest and engagement with this intervention. Finally, a lasting intervention will require clinical champions at primary care sites to help foster interest among treatment staff. If clinical providers are not interested, this will hamper dissemination to patients. The initial qualitative aspect of this trial, which focuses on facilitators and barriers, will be pivotal in maximizing the potential long-term success of this intervention.

### Conclusions

Addiction is widely prevalent and negatively impacts health from both acute and chronic use. Treatments are available; however, uptake is low, and access is limited. Senyo Health is a digitally native platform that combines multiple evidence-based interventions to address and treat SUDs. Its flexibility is particularly useful for rural populations, who often experience severely limited access to treatment. Senyo Health is freely available for download to be used without CM, LADC, or CoCM components. However, we feel that these aspects are essential for the highest treatment efficacy.

## Appendix

Multimedia Appendix 1 Semistructured interview used in the clinical trial.

## Abbreviations

AUDIT-C: Alcohol Use Disorders Identification Test-Consumption
CBT: cognitive behavioral therapy
CFIR: Consolidated Framework for Implementation Research
CM: contingency management
CoCM: collaborative care management
DAST: Drug Abuse Screening Test
HIPAA: Health Insurance Portability and Accountability Act
IRB: institutional review board
LADC: licensed alcohol and drug counselor
SBIRT: screening, brief intervention, and referral to treatment
SUD: substance use disorder

## Data Availability

The datasets generated or analyzed during this study are not publicly available due to patient confidentiality protections and institutional data-sharing policies but are available from the corresponding author on reasonable request.
