# Supplementary material for: Treatment of Substance Use Disorders With a Mobile Phone App Within Rural Collaborative Care Management (Senyo Health): Protocol for a Mixed Methods Randomized Controlled Trial
Source: JMIR Res Protoc. 2025 Mar 26;14:e65693. doi: 10.2196/65693 (PMC11982759; doi:10.2196/65693)
Supplement: Multimedia Appendix 1 [file resprot_v14i1e65693_app1.pdf]

Senyo Semi-structure Interview Questionnaire

Professional participant section

Greeting:
Hi and thank you for agreeing to talk with us today. My name is … and this is my colleague (if applicable) [introduce interviewer 2].

We are researchers at in the Kern Center at Mayo and today we would like to talk with you about your use of the Senyo app and web-based portal. Our goal is to identify specific things that have stood out as helpful to you in managing your patients with chemical dependency. There are no right or wrong answers to these questions and the information that we collect will be used to improve the app and portal.

Consent:
If necessary, remind them of the previous consent and confirm signed HIPAA form received.

Permission to Record:
Do I have your permission to record this interview? If yes: Great, thank you, I am going to begin recording now and I will ask you that question again after I begin for our records.

[Begin recording.]

Once again for the record, do I have your permission to record our discussion today?

Thank you, we are ready to begin. Do you have any questions for us before we start the interview?

Background Information on Respondent:
As I mentioned, our discussion today will be focused on the Senyo app and portal. First it would be helpful to understand your background in caring for patients with CD.

Would you mind telling us about your role at Mayo and history of caring for patients with CD?

What were the main challenges that you encountered in managing your patients with CD?
    Are there specific and predictable periods of time that are more or less difficult for your patients to adhere to their care plans?

What are the most common challenges your CD patients face in maintaining their health?

What are the most common comorbidities among your CD patients?
    How do these conditions impact their care management plans and adherence to care?

Demographic:
What is your age?
What gender do you identify as?

Health Care Access and Utilization:
Why do you think that your patients come to Mayo for CD care?

In your opinion, what could be done to cut back on avoidable utilization, such as an ED visit, related to substance use among your CD patients?

Do your CD patients ever avoid regularly scheduled or needed care? (probe for common reasons)

Care Management:
OK now I would like to shift to more focused questions about health-related apps for CD patients. We are working with a team at Mayo to improve the Senyo app for patients as well as providers like yourself and so I am going to start with a few questions about your experience with that specific app and portal.

What was the most helpful aspect of the app with respect to patient care? (probe for direct impact on health care utilization, connection to patient via messaging, patient self-management in between clinic visits, etc)

Has Senyo changed how you interact with your CD patients?

Do you think Senyo would have an impact on their physical or mental health? (probe for specific impacts)

How has it impacted the ways in which they care for themselves in between visits to the clinic?

App Usability:
We have talked about the impact of the app on your patients' health, but now I am going to ask you about your experience with the app itself – things that you liked or didn't like about using it.

Is the app easy to use?

How often do you think patient would you use this app?

What prompts you to open the app? (probe for specific examples/activities/thoughts)

How long would you use this app if you were a patient?

Do you think patients would switch from this app to another app you are currently using?

What features should be added, removed, or changed?

What additional problems or challenges would you like the app to help your patient with ?

Was the app reliable? (probe for whether or not they experienced any bugs or problems)

App Acceptability:
What did you like most about the app?

If they have used another app in the past: How does Senyo compare to the other app(s) you have used? (probe for things they like better, things that are worse/missing, etc

Do you think that you will continue using the app? (probe for why or why not)

Can you think of any other changes to the app that should be made to improve the experience of using the app?

Portal Usability:
Now I would like to ask about your experience using the web-based portal associated with the app. You would have accessed this on a computer or mobile device browser just like any other website in order to monitor patient usage of the app as well as to communicate with patients.

Did you find the portal easy to use? (probe for why or why not)

Do you think this portal would be helpful for you or your patients in CD treatment? (probe for specifics)

Portal Acceptability:
What did you like about the portal?

What did you dislike about the portal?

In your opinion, was there important information or functionality missing from the portal?

Wrap-up:
OK, we have asked all our questions. Is there anything else that you would like to discuss related to the topics we have covered?

Thank you so much for taking the time to talk with us and have a great day!

[Turn off the recording device.]

<u>Patient participation section</u>

Greeting:
Hi and thank you for agreeing to talk with us today. My name is … and this is my colleague (if applicable) [introduce interviewer 2].

We are researchers at Mayo Clinic and today we would like to talk with you about your experience receiving care at Mayo. Our goal is to identify specific things that have stood out as helpful to you in maintaining your health in between visits to the clinic, as well as some things that could have been done better or were missing altogether. There are no right or wrong answers to these questions and the information that we collect will be used to improve care for patients like yourself.

Consent:
If necessary, remind them of the previous consent and confirm signed HIPAA form received.

Permission to Record:
Do I have your permission to record this interview? If yes: Great, thank you, I am going to begin recording now and I will ask you that question again after I begin for our records.

[Begin recording.]

Once again for the record, do I have your permission to record our discussion today?

Thank you, we are ready to begin. Do you have any questions for us before we start the interview?

Demographic information:
What is your age?
What gender do you identify as?
What has been or is your drug of choice?
How many treatments for substance use have you attended (include inpatient & outpatient)?

Background Information on Respondent (treatment history):
As I mentioned, our discussion today will be focused on what Mayo has done and could be doing better to help you manage your health in between visits to the clinic. We are particularly interested in the care that you have received related to your substance use disorder.

When were you first diagnosed with a substance use disorder?
- About how old were you at the time of diagnosis?
- Where were you diagnosed? (i.e., Mayo vs elsewhere)
- What substance(s) are you seeking care for?

How has your SUD impacted your health?
- What were the main challenges that you encountered in managing your health during the first year after diagnosis?

What are your biggest challenges to staying healthy now?

Have you ever been diagnosed with any other chronic conditions? (i.e., beyond substance use disorder)
- What are the main challenges you face in managing that condition (those conditions)?
- How does this impact, if at all, your health related to your substance use disorder?

Health Care Access and Utilization:

How long have you been receiving care for your substance use disorder at Mayo Clinic?
Why did you start coming to Mayo?
Do you feel like you have a care team at Mayo?
What do you like the most about the care you receive from Mayo?

Since your diagnosis, has there ever been a time in which you ended up in the emergency department because of your substance use? (probe for how that could have been avoided)

Since your diagnosis, has there ever been a time in which you needed help from your health care team but didn't receive it? (probe for why they didn't seek care or if care was not accessible – lack of insurance, transportation issues for rural residents – and how that could have been avoided)

Care Management:
Have you ever used an app to help manage your health related to your substance use disorder?
If yes:
Which apps have you used?
When did you start using [app]?

We are working with a team at Mayo to improve the Senyo app for patients like yourself and so we are going to ask some questions about your experience with that specific app.

Why did you begin using Senyo?
How do you use it, if at all, in your day-to-day life?
What is the most helpful aspect of the app? (probe for direct impact on health care utilization, connection to care team, self-management in between clinic visits, etc)

Has Senyo changed how you interact with your health care providers at Mayo? (probe for specific features and impacts)

Have you noticed whether your use of Senyo has had an impact on your physical or mental health? (probe for specific impacts)
How has it impacted the ways in which you care for yourself in between visits to the clinic?

Usability:
We have talked about the impact of the app on your health, but now I am going to ask you about your experience with the app itself – things that you liked or didn't like about using it.

Is the app easy to use?

How often would you use this app?

What prompts you to open the app? (probe for specific examples/activities/thoughts)

How long would you use this app? Do you think you would use this app more than a week? More than a month? More than 6 months?

Would you switch from this app to another app you are currently using?

What features should be added, removed, or changed?

What additional problems or challenges would you like the app to help you with?

Was the app reliable? (probe for whether or not they experienced any bugs or problems)

Do you feel the app is secure? Do you have any concerns about having sensitive information on the app?

Acceptability:
What did you like most about the app?
If they have used another app in the past: How does Senyo compare to the other app(s) you have used? (probe for things they like better, things that are worse/missing, etc

Do you think that you will continue using the app? (probe for why or why not)

Can you think of any other changes to the app that should be made to improve the experience of using the app?

Wrap-up:
OK, we have asked all our questions. Is there anything else that you would like to discuss related to the topics we have covered?

Thank you so much for taking the time to talk with us and have a great day!

[Turn off the recording device.]
